# Analysis of ^14^C, ^13^C and Aspartic Acid Racemization in Teeth and Bones to Facilitate Identification of Unknown Human Remains: Outcomes of Practical Casework

**DOI:** 10.3390/biom11111655

**Published:** 2021-11-08

**Authors:** Rebecka Teglind, Irena Dawidson, Jonas Balkefors, Kanar Alkass

**Affiliations:** 1 Department of Oncology-Pathology, Karolinska Institute, 171 77 Stockholm, Sweden; rebecka.teglind@ki.se; 2 Department of Forensic Medicine, The National Board of Forensic Medicine, 171 77 Stockholm, Sweden; irena.dawidson@rmv.se; 3 Tandem Laboratory, Ångström Laboratory, Uppsala University, 751 20 Uppsala, Sweden; jonas.balkefors@physics.uu.se

**Keywords:** human remains, identification, forensic medicine, date of birth, date of death, carbon-14, stable isotopes, aspartic acid racemization

## Abstract

The identification of unknown human remains represents an important task in forensic casework. If there are no clues as to the identity of the remains, then the age, sex, and origin are the most important factors to limit the search for a matching person. Here, we present the outcome of application of so-called bomb pulse radiocarbon (^14^C derived from above-ground nuclear bomb tests during 1955–1963) analysis to birthdate human remains. In nine identified cases, ^14^C analysis of tooth crowns provided an estimate of the true date of birth with an average absolute error of 1.2 ± 0.8 years. Analysis of ^14^C in tooth roots also showed a good precision with an average absolute error of 2.3 ± 2.5 years. Levels of ^14^C in bones can determine whether a subject has lived after 1955 or not, but more precise carbon turnover data for bones would be needed to calculate date of birth and date of death. Aspartic acid racemization analysis was performed on samples from four cases; in one of these, the year of birth could be predicted with good precision, whereas the other three cases are still unidentified. The stable isotope ^13^C was analyzed in tooth crowns to estimate provenance. Levels of ^13^C indicative of Scandinavian provenance were found in known Scandinavian subjects. Teeth from four Polish subjects all showed higher ^13^C levels than the average for Scandinavian subjects.

## 1. Introduction

Dead victim identification constitutes an important task for forensic professionals including forensic pathologists, anthropologists, and odontologists in their daily casework, particularly in mass disasters. If there are no clues as to the identity of the deceased, age, sex, and origin represent particularly important information to limit the search for possible matching persons. Whereas the sex often can be determined by morphological characteristics, or by DNA analysis, the age of adult subjects is more difficult to estimate. Since structural parts of teeth and bones are highly resistant to decomposition, chemical degradation, and heat, they may constitute the only material available for forensic analyses. Many different methods for estimating the age of a deceased person have been published, most of them based on examination of teeth and bones. Anthropological analyses based on morphological changes typically provide age estimation errors for adults of ±10 years [1,2,3,4,5,6], which imply intervals that are too wide to offer meaningful help in practical casework. A number of chemical and molecular analytical methods have therefore been developed to improve the accuracy of predicting the age of deceased subjects [1,2,7,8,9,10,11,12,13,14,15,16,17]. One of the most useful chemical methods to assess age is aspartic acid racemization (AAR) analysis, which is based on the gradual increase in the D-form of aspartic acid in tissues with age. Virtually all amino acids undergo conversion from L-form to D-form with time, and the extent of racemization of amino acids can be used to estimate the age of various tissues [18,19]. Of all stable amino acids, aspartic acid has one of the fastest racemization rates and is, therefore, the amino acid most frequently used for age estimation. Tissues with low metabolic rates, such as tooth enamel and dentin, are most suitable for analysis. Hence, AAR for the purpose of age estimation is most often performed on tooth dentin, and some studies have reported mean average errors as low as 2–4 years [7].

In practical casework, the primary focus is to determine whether the find is of human origin, and then whether it is recent or old, warranting a further police investigation or not. One powerful technique that is useful in this respect is the retrospective radiocarbon dating of modern biological material [20,21]. In forensic casework, the estimation of time of death is particularly important since it may be decisive as to whether the police should investigate or close the case. [22,23].

**Figure 1:**
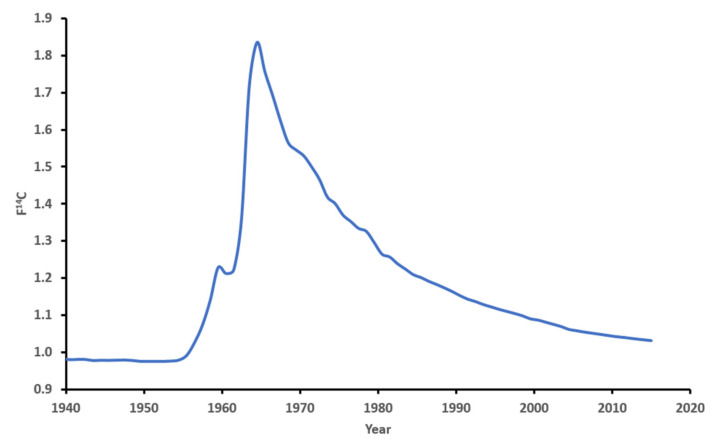
Average annual atomspheric ^14^CO_2_ records for Northen Hemisphere.

Carbon-14 (^14^C) is naturally formed in the atmosphere by cosmic rays’ interactions with nitrogen-14 (^15^N). Carbon atoms in the atmosphere are quickly oxidized to form CO_2_, which is then incorporated into plants via photosynthesis. The ^14^C levels in the atmosphere have stayed fairly stable for several thousands of years. However, atmospheric above-ground detonations of nuclear weapons during the Cold War (1955–1963) almost doubled the concentration of ^14^C levels in the atmosphere, which was rapidly distributed around the globe [24,25]. This is known as the “bomb curve”, which reached its maximum concentration in 1963 (Figure 1). Since 1963, because of a worldwide test ban treaty, the ^14^C levels in the atmosphere have been decreasing exponentially. This decrease is not due to radioactive decay (^14^C has a half-life of 5730 years) but is caused by mixing with large marine and terrestrial carbon reservoirs.

The bomb pulse-generated artificial variation in atmospheric ^14^C levels has offered an opportunity to birthdate the formation of biological material using sensitive accelerator mass spectrometry (AMS) analysis. We have taken advantage of this by analyzing ^14^C in tooth enamel, which does not exchange any carbon atoms with the environment after it has been formed. In their pioneering study, Spalding et al. [26] reported a mean absolute error of 1.6 ± 1.3 years in predicting the year of birth from ^14^C analysis of tooth enamel. Similar precision has subsequently been reported in several studies [27,28,29,30]. Alkass et al. further provided reference data for ^14^C incorporation times in tooth enamel, which are more practical to use than radiographic laydown times for the calculation of a person’s date of birth [31]. The limitation of bomb pulse radiocarbon analysis is that it cannot provide any useful estimates if the analyzed material was formed before 1955, given that nuclear bomb tests had not yet increased atmospheric ^14^C levels. Further, analysis of tissues with a high turnover—virtually all soft tissues except the lens of the eye [32]—will not provide a good estimate of the person’s year of birth or year of death, but rather a year in between, which will vary depending on the particular tissue’s carbon turnover rate. Another future problem is that the atmospheric levels are approaching baseline, meaning that the application of the method on subjects born today or later will not show such excellent precision.

In archeological casework, the collagen fraction of bone [33] is preferred for the estimation of year of death since it is less affected by external contamination of carbon. Collagen makes up a large proportion of the protein content of the skeletal tissue and is easy to extract. Since there is a turnover of collagen in the bones, the ^14^C levels will match times in between the birth and the death of the individual. For archeological studies, this is not a critical issue, but when dating post-bomb skeletal remains in criminal investigations, it is certainly important to be aware of the variation in levels that may be found in different types of bones of persons of varying ages [34,35].

In addition to radiocarbon dating of tooth crowns, it is also possible to analyze stable isotopes in tooth enamel and dentin to obtain clues about the provenance [28]. Although commercially available genomic tests may disclose ancestry, the geographical place where a person was born and/or raised may be quite different. Analysis of stable isotopes that show a geographical and/or cultural variation can provide more useful information. For instance, the levels of the isotopes ^2^H and ^18^O vary with latitude and precipitation, which in part are associated with the distance from coastal areas, and will show geographical variation, reflected in the drinking water. Hence, analysis of these isotopes in teeth will give a rough idea of the geographical region where a person was raised. In contrast, ^13^C levels reflect the diet, which partly depends on geography and partly on cultural habits. So-called C4 plants, which include crops that grow in hot areas (e.g., corn and sugar cane), enrich ^13^C due to a different CO_2_ assimilation as compared to so-called C3 plants (which include rice, potato, and beet root), which thrive in colder climate regions [36,37]. We have previously reported that teeth from subjects raised in different countries contain region-dependent variations [28,31].

Today, there are numerous publications on a variety of methods for age estimation of human remains, but there is a paucity of reports regarding the performance of these methods in practice. Hence, we sought to study the panorama of cases in which the police requested investigation regarding age, sex, and provenance, and to what extent the bomb pulse ^14^C method, AAR analysis, and ^13^C analysis could provide helpful information.

## 2. Materials and Methods

### 2.1. Examined Samples

The material studied consisted of teeth and bones from human subjects. The remains were obtained from the six Swedish forensic medicine departments, which in turn had received them from the police during the period 2011–2021. Table 1 provides an overview of the cases in which the police had requested further analysis regarding age, sex, and origin. For each case, we inquired about which parts of the material were available and asked the responsible pathologist which bones or teeth could be used for our analysis. Together with forensic odontologists, permanent teeth with the least or no caries, no restorations, no endodontic treatment, and a complete crown were selected, if available. The tooth number and the condition of the tooth were recorded as well as gender, if known. For cases lacking teeth, we used cortical bone from the bones that were available, which most often were calvarial bones or long bones, from the extremities. The police had opened a homicide investigation in all cases where the find was a whole body, and in several of the other cases as well (where partial remains were found).

### 2.2. Tooth Preparation

The soft tissue and dirt were removed mechanically using an odontological electric drill. The tooth crown was cut away from the root at the level of the cervical line. The crown and root were then incubated in 0.2 N HCl at room temperature in a sonicator water bath (Branson 150 GIMA, Gessate, Milano, Italy).The crown and root were washed three times with ddH_2_O and placed again into the sonicator water bath. This procedure was repeated three times, and the material was then dried at room temperature. Next, the entire crown and root were crushed using Qiagen Tissue-lyser II. A fine powder was obtained using a standard program with a run time of 30 s. The powder was then further pre-treated at the AMS laboratory.

### 2.3. Sample Pre-Treatment and AMS Analysis

Sample pre-treatment was performed on all samples prior to AMS analysis. The carbonate (mineral) fraction was analyzed for both tooth crown and root samples. In some cases, the collagen fraction was also analyzed in tooth roots to allow for comparison between the isotopic levels in the two fractions. For bone samples, only the collagen fraction was analyzed. Procedures for the extraction of each fraction are described below.

### 2.4. CO_2_ Extraction of Carbonate Fraction from Tooth Crown and Root

In this step, 10 mL 0.8 M HCl cooled to 5 °C was added to 200–500 mg of the tooth sample in a low vacuum reaction chamber. The subsequent leaching reaction was carried out at room temperature for a duration of at least 1 h until no further gas release was observed. The obtained CO_2_ was purified, split, cryogenically trapped, and finally reduced by graphitization with an iron catalyst [38,39].

### 2.5. Collagen Extraction from Bone and Tooth Root

Collagen extraction was performed by a standard HCl–gelatin procedure [40]. Sample pieces were cut mechanically from the bones, and the top layer was removed to eliminate possible contaminants from the surface. The sample pieces were then powdered in a mortar. Next, 100–500 mg of the bone sample was first de-mineralized by removing the apatite fraction with 0.8 M HCl at 10 °C until the evolving gases stopped after 30–60 min. The insoluble fraction was rinsed with de-ionized water to a neutral pH. Diluted HCl was added to the insoluble fraction and held at pH 3 for 6–8 h at 90 °C. The soluble fraction from this extraction contains the fraction with the highest amount of collagen and was dried and combusted. The obtained CO_2_ was split, purified, trapped, and reduced by graphitization with an iron catalyst [38,39].

### 2.6. AMS Analysis and Isotope Ratio Mass Spectrometry

The ^14^C AMS analysis was performed on the graphitized samples at the Tandem Laboratory, Uppsala University, using the 5 MV pelletron tandem accelerator or the MICADAS (mini radiocarbon dating system) [41,42]. Calibrated ages on the samples were obtained from the Oxcal website [43]. A small portion of the obtained CO_2_ that was split prior to graphitization was used to perform ^13^C and ^15^N stable isotope measurement using isotope ratio mass spectrometers, (VG OPTIMA dual inlet MS and Elementar precision IRMS, Isoprime Inc, Manchester, UK).

### 2.7. Determining Year of Birth from ^14^C Analysis: Teeth

The average age at which the formation of tooth crowns and roots is completed for each specific tooth has been determined previously and is dependent on the tooth number and gender. We used the formation times for tooth roots reported by Nolla [44], but for the enamel we used our own reference data for ^14^C incorporation times [31]. For the translation of tooth and bone ^14^C levels to matching atmospheric levels, we used the CALIbomb Levin data set [43] with 1.0 years smoothing and 2 σ error.

### 2.8. Selection of Cases for Aspartic Acid Racemization (AAR)

AAR analyses were performed on nine teeth from four cases to provide an estimate of the person’s age at death. These results were used to estimate the year of death in three cases, in which the year of birth had been estimated by ^14^C analysis, and conversely, to estimate the year of birth in one case with a known date of death.

### 2.9. Method Protocols for AAR Analysis

In Case 2, the AAR analysis was performed according to a previously described protocol [45]. Briefly, 1 mm-thick median longitudinal sections were made by cutting the teeth with a low-speed cutter (Isomet, 11-1180, Buehler, Chicago, IL, USA). After cleaning procedures, the dentin sections were pulverized in an agate mortar, and 10 mg of the powder was used as the specimen for determination of the racemization ratio. The D- and L-forms of aspartic acid were measured by gas chromatography using a glass capillary (GC-17A, Shimadzu, Kyoto, Japan) after hydrolysis and derivatization. The column of the glass capillary was 30 m in length, 0.3 mm in internal diameter, and coated with Chirasil-Val (GL Science, Tokyo, Japan). The concentration of the D-form was related to the concentration of the L-form, and the racemization ratio was expressed as ln((1 + D/L)/(1−D/L)). Plotting the chronological age on the *x*-axis and the racemization ratio on the *y*-axis, we derived the following linear regression equation by the least squares method:

ln[(1 + D/L)/(1 − D/L)]*_t_* = 2*kt* + ln[(1 + D/L)]*t* = 0 (1)

where ln ((1 + D/L)/(1 − D/L)) represents the log-transformed racemization ratio, *t* is the chronological age, and *k* is the racemization rate constant. To estimate the chronological age, we plotted the age against the racemization ratio and derived the following linear regression equation by the least squares method.

*t* = {ln[(1 + D/L)/(1 − D/L)]_t_ − ln[(1 + D/L)/(1 − D/L)]_t = 0_}/2*k* (2)

The estimated age was obtained by substituting the D/L ratio in this linear regression equation with that of the specimen to be estimated. For a detailed flowchart illustrating the method of racemization analysis, see [46].

Cases 14 and 16 were analyzed at the forensic toxicology laboratory in Linköping, Sweden. The analysis was performed on 1 mm-thick median longitudinal sections, prepared, cleaned, and pulverized as described above. The aspartic acid enantiomers were measured by (Nexera UHPLC Shimadzu, Stockholm, Sweden) with mass spectrometric detection, after hydrolysis and derivatization. The chiral column Astec Chirobiotic T 2, 1 × 250 mm, 5 μm was used. The concentrations of the D- and L-forms were determined, and the racemization ratio was expressed as above. Case 28 was analyzed at the Department of Legal Medicine, University Hospital, Düsseldorf, Germany, by gas chromatography with flame ionization detector according to a previously described protocol [47]. Briefly, horizontal rings of the roots beneath the cervical line were prepared. After careful cleaning steps and isolation of dentin, the samples were pulverized with a hydraulic press (P/O/Weber, Remshalden, Germany) at 20 kN. Next, the pulverized dentin was hydrolyzed in 1 mL 6 N hydrochloric acid for 6 h at 100 °C and dried in a vacuum desiccator. For derivatization, 1 mL isopropanol and 1 μL sulfuric acid were added to each sample, and the samples were heated at 110 °C for 1 h. After adding 1 mL 4 N ammonia solution and 1 M dichlormethane, the samples were centrifuged, and the resulting two phases were separated and dried again. Then, 1 mg dichlormethane and 50 μL trifluoroacetic acid (TFAA) were added. The samples were then heated for 15 min at 60 °C and dried using a nitrogen stream. Separation and quantification of D- and L-aspartic acid were performed on a chiral capillary column in a gas chromatograph (GC Shimadzu GC-2014, Agilent Technologies, Santa Clara, CA, USA). Column: Chirasil-L-Val, Varian, (Agilent Technologies, Santa Clara, CA, USA). A defined sample of aspartic acid with a known D/L ratio (Merck, Darmstadt, Germany) was used as a standard for quality assurance. Each sample was analyzed at least twice. The extent of aspartic acid racemization was calculated as ln((1 + D/L)/(1−D/L)) [47].

### 2.10. DNA Analysis

DNA analysis was performed at the Department of Forensic Genetics, Swedish National Board of Forensic Medicine, Linköping, Sweden. The IdentiFiler^TM^ and GlobalFiler^TM^ STR kits (ThermoFisher Scientific, Waltham, MA, USA) were used according to the manufacturer’s instructions.

### 2.11. Statistical Analysis

All statistical analysis was carried out using SPSS version 25 (IBM SPSS Inc., Chicago, IL, USA). Results are expressed as mean ± SD. Linear regression analysis was used to calculate the correlation between the estimated and true date of birth.

## 3. Results

Table 1 shows the characteristics of the material (tissue) used and the methods applied in each instance. Sixty-three teeth from a total of 34 individuals, and 24 bones from 21 individuals were analyzed.

### 3.1. Bomb Pulse ^14^C Dating of Tooth Crown Precisely Determines Date of Birth

**Figure 2:**
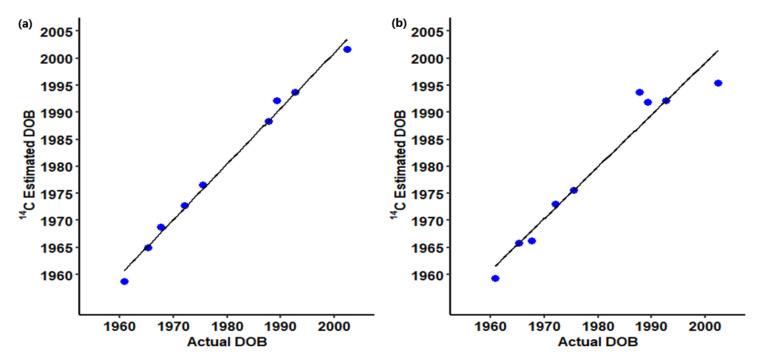
Linear regression analysis of (**a**) the estimated date of birth (dob) based on ^14^C analysis of tooth crown and actual dob (R^2^ = 0.991). (**b**) the estimated dob based on ^14^C analysis of tooth roots and actual dob (R^2^ = 0.941).

Out of the 34 cases, 18 cases were found to be post-bomb, i.e., the tooth crown was formed later than 1955 (Table 2), allowing ^14^C bomb pulse dating. Nine of these 18 cases were eventually identified, and a high correlation was found between the estimated and true year of birth for these individuals with an average absolute error of 1.18 ± 0.83 years (R^2^ = .991, n = 9; Figure 2a). The ^14^C levels were consistently higher in the root than the crown if the crown was formed during the rising part of the bomb curve and were lower if the crown was formed around the peak or later. In the remaining nine cases, the identity has still not been established; these comprise two old Norwegian cases, four Swedish homicides where the investigation was still ongoing, and three cases that were closed.

In 25 cases, the tooth crowns of two or more teeth were analyzed from the same individual, with different tooth formation times. This allowed us to determine whether the values obtained match the rising or the falling part of the bomb curve [28]. The average of these two values was used to estimate the date of birth of the individual. To this end, we subtract the average ^14^C incorporation time [31], instead of the radiological formation time, yet the latter can provide a good correlation between the estimated and true date of birth of the individuals (absolute average error 1.3 ± 1.2 years, R^2^ = 0.987). Even if the person was born before the onset of the bomb curve, there is a possibility of observing positive ^14^C readings in the teeth: For the third molar in the upper jaw, the crown and the root are formed on average 11.7 and 17.0 years, respectively, after birth. For instance, in Case 33, tooth 43 showed a pre-bomb ^14^C level in the crown, but the root displayed a post-bomb value and allowed for an estimate of the person’s year of birth. This particular case is still under investigation and the identity not yet established. We also found a high correlation between the estimated age based on ^14^C levels in tooth roots and the actual age of the subject with an average absolute error of 2.3 ± 2.5 years (R^2^ = 0.941, n = 9, Figure 2b).

### 3.2. ^14^C Analysis of Bones

Table 3 summarizes the information about the 21 deceased persons from whom bone collagen was extracted and analyzed, and in eight cases, AMS analysis revealed post-bomb ^14^C values. From macroscopical examination of the remaining 13 cases, the police or forensic pathologist either concluded that the bones were modern or at least could not exclude this possibility. However, each of these cases provided pre-bomb ^14^C values, and given that these values suggested that some were very old (Table 3), the police were able to close the cases.

In two cases (14 and 41), both trabecular and cortical bone was analyzed. In Case 41, the trabecular bone sample showed a higher ^14^C level than the cortical bone sample, which can originate from a person who died during or some years after the peak of the bomb curve. In Case 14, the trabecular bone instead showed a lower ^14^C level than the cortical bone, suggesting that the person had been alive quite some time after the peak of the curve in 1963. In this case, the person was eventually identified and indeed was born in 1965.2, and was reported missing in 2008, but the true date of death had not yet been determined. We subsequently carried out AAR analysis of dentin from two teeth and estimated that the man was 36.7 years old when he died (Table 4). When adding this to the date of birth, we calculated the date of death to be 2001.9. We then also used the standardized lag time for carbon turnover for subjects of this age as suggested by Ubelaker [34], which gave 2001.6 as the estimated year of death.

Case 42 regards a newborn baby who was found severely decomposed in a plastic bag in a garden in 2018. The police suspected a 24-year-old woman to be the mother, and this was soon confirmed by DNA analysis. According to the woman, she gave birth to the child in 2009 (when she was 15 years old), yet this statement was questioned since the body had been lying outdoors and soft tissues remained. Since the baby lacked teeth, we carried out ^14^C analysis on the femur and found levels matching 2008.3, hence supporting the mother’s account. This implied that the woman was not prosecuted, given that she was underaged at the time of the event.

### 3.3. ^13^C in Teeth and Bones Can Tell Geographical Origin

The ^13^C levels in the carbonate fraction of all 114 tooth samples (crowns and roots) from 34 individuals were analyzed with IRMS. All subject information and raw data are listed in Table 2. In four cases (Cases 4, 5, 6, and 8), the ^13^C levels were higher than expected for Scandinavian subjects [28,31]. All four subjects were eventually identified, revealing that these individuals were born and raised in Poland and had average ^13^C levels of −12.7 ± 1.8 compared with −15.9 ± 1.8 for tooth samples from Swedish subjects in this study and −14.9 ± 0.3 for all Scandinavian subjects that we have analyzed previously reported [28,29,31]. In 20 tooth samples, both the collagen and carbonate fractions were measured, and the ^13^C levels averaged −12.6 ± 4.3 and −20.5 ± 4.2 in the carbonate and collagen fraction, respectively.

The ^13^C levels were also measured in the collagen fraction of 24 bones from 21 individuals (Table 3). The levels averaged −20.3 ± 2.3, hence being similar to the levels in the collagen fraction of teeth. The only outliers are Cases 33 and 36, with both cases still under investigation, pending clues as to both cause of death and identity. To assess the possible contamination of the samples, the stable isotope ^15^N was also measured. The ratio ^13^C and ^15^N (C/N) averaged 3.3 ± 0.1, supporting a good quality of the stable isotope samples; the C/N ratio should be in the range of 2.9–3.6 [48].

### 3.4. Aspartic Acid Racemization (AAR) Analysis

AAR was performed on root dentin samples from four cases (Table 4). In Case 14, this allowed for an estimate of the year of death of the person, since the year of birth had been estimated with ^14^C analysis, and this information helped the police to identity the person. The identities of the other three subjects have not as yet been determined. However, since we were able to estimate the year of birth for Cases 2 and 28, the AAR analysis allowed us to calculate the year of death by adding the estimated age at death, which was of particular interest to the police. However, both investigations are still ongoing.

Case 16, named the Isdal woman, has attracted wide interest since her partly burnt body was found in 1970 in a remote spot, Ice Valley (Isdal in Norwegian), near Bergen in Norway [49]. We obtained teeth from this case in 2019 and performed ^14^C analysis on the crowns of three teeth, but all showed pre-bomb values, including tooth 48, which has an average ^14^C incorporation time of 11 years after birth [31]. Hence, we then performed AAR analysis of dentin from three tooth roots to estimate the age at death, which then could be subtracted from the year of death. As seen in Table 4, the roots of teeth 24 and 25 gave a similar age, whereas the frontal tooth 22 gave a much younger age The average of all three teeth gave the estimated date of birth 1932.4, however the case remains unsolved.

### 3.5. DNA Analysis

In 13 cases, a successful DNA profile could be obtained using the Identifiler^TM^ or GlobalFiler^TM^ PCR amplification kits, by which the gender can also be determined. Comparative analysis with these kits was performed in all nine cases that were eventually identified. In a further 12 cases, DNA profiling failed due to poor quality of the DNA. In the remaining cases, a few well-preserved bodies could be visually identified, and in other cases DNA profiling was not considered meaningful since the remains were too old to justify an investigation.

## 4. Discussion

We show here that radiocarbon bomb pulse ^14^C analysis of teeth is a technique that can be used in forensic practical casework to estimate the year of birth with a precision that is just as good as that observed for analysis of teeth extracted from living patients [26,27,28,29,30,31,50]. Concern has been expressed regarding the possibility of carbon contamination of tooth and bone samples from the environment [51]. For the bone samples analyzed, we cannot determine whether any such contamination might have influenced the ^14^C levels since the carbon turnover in the samples is unknown and hence it is not possible to tell which values are to be expected if contamination is absent. Regarding tooth enamel and dentin, however, we found that bomb pulse ^14^C analysis could predict the true year of birth with excellent precision even though several cases had been exposed to soil, air, and water for extended times, hence excluding any major degree of contamination of the teeth.

By cutting off the crown, removing dentin remnants, and then pulverizing the enamel mechanically with a bead mill, we could dramatically expedite the standard extraction procedure previously reported [28] while maintaining a high prediction precision. Originally, we treated the enamel with 10 N NaOH in an ultrasonic bath for one day, then removed non-enamel remnants and cleaned the samples, and again re-submerged them in new NaOH every 24 h for up to ten days [26,29]. Then we tried cryo-crushing the teeth in liquid nitrogen, but even if this technique provides a suitable powder in ten minutes, it requires liquid nitrogen to be available at any time needed. The Tissue-Lyser II offers a pure mechanical pulverization, and by optimizing the amplitude and frequency, we determined settings that produce a fine powder in 30 s. Hence, with this pulverization and cleaning and drying steps, enamel can typically be isolated within one day, and if the AMS lab has the opportunity to analyze the samples soon after arrival, results may be obtained in less than a week.

We also show that radiocarbon dating of tooth roots provides estimates of year of birth nearly as good as enamel, with no systematic deviation in the prediction of an individual’s date of birth. This suggests that dentin, even though it is considered a “living tissue”, has a very low turnover of carbon, otherwise we would have obtained estimates indicating a more recent year of birth for the subjects studied. This is in agreement with the results of AAR analysis of dentin, which requires a low turnover in order for the D-aspartic acid to be formed [52]. Roots are better preserved and less prone to odontological diseases than enamel, and therefore may represent a feasible alternative to enamel for birth dating. In addition, since tooth roots are formed later than the enamel, analysis of tooth roots can increase the chances of obtaining a positive signal in subjects born earlier before the onset of the bomb curve.

Compared with tooth enamel and dentin, bone tissue is continuously remodeled, implying a turnover of carbon at a pace that makes dating the birth or death of a person from ^14^C analysis in bone difficult. There are up to 220 different types of bones in the body, and their carbon turnover rates are not well characterized. Ubelaker et al. [20] have provided radiocarbon lag times for subjects of different ages to calculate date of death. However, their reported lag time averages for different decades of age of the individuals varied from 11.9 to 33.6 years. Hence, if the age of the individual is very uncertain, the calculation of date of death will result in a wide time window if reference lag times for several decades of life have to be used. If an estimate of year of birth is indeed available, such as from a post-bomb result of teeth, or if a method for estimating age at death has been employed, such as AAR or DNA methylation analysis, then the pertinent lag time may be used. Even more attractive, however, is the option of using reference carbon turnover data for different bones or different extraction fractions of bones and applying similar mathematical modeling used previously to calculate the turnover of various cell populations [53,54,55]. To obtain such reference data, analysis of fractions of select bones from subjects with known dates of birth and dates of death is required, which will take time. Hence, at present, a post-bomb ^14^C value suggests that the person was alive after 1955, whilst a pre-bomb value suggests that the person either was not alive after 1955 or died soon after that. If the ^14^C values are very high, it may be deduced that the person died within a decade or so after the bomb peak. In Case 47 (Table 3), the levels were very high, and it is obvious that the person must have lived through the bomb peak period, but most likely died not many years after that. If a find includes nails or hair, ^14^C analysis of these can provide an estimate of date of death with a good precision as they will show contemporary values [35,56,57]. When it comes to children, the age at death is usually fairly easy to estimate from morphological features, and regarding infants, such as Case 42, any tissue can be used for ^14^C dating since they will all have been formed within a short time window.

We have previously reported that combined AAR and AMS ^14^C analyses can be performed on teeth to provide information about both the year of birth and year of death of an individual [29]. AAR analysis of dentin provides information about the chronological age of an individual at death because the chemical conversion from the L-enantiomer to the D-enantiomer will typically stop completely after death, unless the body has been lying in a hot environment. AAR will, however, not inform us about the calendar year of birth (or death). To determine these calendar years, other methods must be used: either a ^14^C birth dating or an estimation of the postmortem interval. One advantage of AAR analysis is that it is independent of the bomb peak and hence can be used for age determination of subjects born long before the beginning of above-ground nuclear weapon testing. Several factors, however, will affect the precision of this method; as the racemization process is effectively a function of temperature and time, the precision varies depending on the tooth type analyzed, most likely due to their exposure to different ambient temperatures [58]. In Case 16 (the Isdal woman), all teeth had been exposed to a fire that could have affected the racemization ratio, and hence the age estimation. However, if the fire had influenced the racemization process, a higher ratio would have been expected for tooth 22 compared with teeth 24 and 25 positioned further back.

Studies on hair samples from subjects from different countries [59] have shown that stable isotopes can display geographical signatures. Similarly, we have reported geographically different ^13^C levels in teeth extracted from patients in many different countries [28,31]. In the present study, most subjects showed carbonate fraction levels around −15, which we previously have found in teeth from known Scandinavian persons. Interestingly, the ^13^C levels in teeth from subjects raised in Poland all had higher levels. When assessing people on the American continent, the high ^13^C levels result from a traditional dietary intake of products from C4 plants, such as corn and sugar cane, but for people living in continental Europe, no such reference ^13^C data or suggested explanation for the elevated concentrations exist. Case 18 was born in Vietnam but raised in Sweden and showed, on average, a ^13^C concentration of 14.6 [37,60]. The ^13^C levels in tooth crowns are shown in Table 2.

Finally, it should be noted that ^13^C levels are different in the carbonate and collagen fractions due to variable fractionation in biological tissue structures [61]. For Scandinavians, the δ^13^C in the collagen fraction will be approximately −20 while the levels in the carbonate fraction will show a higher average, around −15, irrespective of whether the collagen is extracted from teeth or bone. Whilst considerable data detailing carbonate δ^13^C in teeth exist from different countries [28,31], no similar reference information exists for the collagen fraction. From the levels measured in the carbonate and collagen fractions of the same samples (Table 1 and Table 2), it is, however, likely that a standard difference may be applied to collagen levels to then approximate the levels in the carbonate fraction; we found an average difference of −7.9 between these fractions in the 20 tooth samples with parallel analysis.

In order to exclude the possibility that external contamination had affected the ^13^C levels, we also measured the ^15^N concentrations and calculated the C:N ratio, which should range between 2.9 and 3.6 in collagen samples from bone [62]. For all bone samples analyzed, the C:N ratio fell within this range, averaging 3.3 ± 0.1.

## 5. Conclusions

We show that radiocarbon dating of tooth enamel provides a very precise estimation of an individual’s date of birth and that very small amounts are sufficient for analysis. AMS determines the date of tooth formation, whilst AAR determines age of death. The combination of these methodologies offers considerable power to forensic pathologists and police authorities to help determine the identity and time of death of unidentified individuals. We expect an increased use in this strategy for as long as the bomb pulse ^14^C levels still remain elevated in the teeth of the population of interest, which implies two or three decades to come.

## Figures and Tables

**Table 1:** Demographic information for the data used in the analysis of F^14^C, δ^13^C and AAR.

Case	Sex ^1^	Body Parts ^2^	Tissue Analyzed	Analyses ^3^	Environment	Remains Found	Identity Established	DNA Profiling
		***Teeth available***						
1	F	Whole body	Teeth 16, 17, 46	^14^C, ^13^C	Fire victim	2012	Yes	Yes
2	M	Whole body, skeletonized	Teeth 15, 27	^14^C, ^13^C, AAR	Air	2013	No	Yes
3	M	Mandible	Teeth 34, 35	^14^C, ^13^C	Water	2013	No	Yes
4	M	Whole body, skeletonized	Teeth 35, 43	^14^C, ^13^C	Air	2013	Yes	Yes
5	M	Cranium	Teeth 13, 35, 47	^14^C, ^13^C	Air	2015	Yes	Yes
6	F	Whole body	Teeth 23, 34, 42	^14^C, ^13^C	Water	2015	Yes	Yes
7	NA	Mandible	Teeth 46, 47	^14^C, ^13^C	Air	2015	No	Yes, but failed
8	M	Whole body, skeletonized	Teeth 14, 17	^14^C, ^13^C	Soil	2015	Yes	Yes
9	NA	Cranium	Teeth 13, 17	^14^C, ^13^C	Soil	2015	No	
10	(F)	Incomplete body, skeletonized	Teeth 14, 21	^14^C, ^13^C	Air	2015	No	
11	(F)	Several bones	Teeth 13, 15	^14^C, ^13^C	Air	2016	No	
12	NA	Several bones	Teeth 34, 38	^14^C, ^13^C	Water	2016	No	Yes, but failed
13	NA	Cranium	Teeth 16, 24	^14^C, ^13^C	Soil	2017	No	Yes, but failed
14	M	Several bones	Teeth 18, 33, calvarium, humerus	^14^C, ^13^C, AAR	Water	2017	Yes	Yes
15	NA	Cranium	Teeth 21, 38	^14^C, ^13^C	Air	2020	No	
16	F	Whole body	Teeth 25, 33, 48	^14^C, ^13^C, AAR	Air, fire	1971	No	Yes
17	F	Whole body	Teeth 31, 34	^14^C, ^13^C	Air, indoor	1995	No	Yes
18	NA	Several bones	Teeth 17, 33, 34	^14^C, ^13^C	Water	2018	Yes	Yes
19	NA	Several bones	Teeth 24, 27	^14^C, ^13^C	Air	2018	No	
20	NA	Mandible	Teeth 38	^14^C, ^13^C	Air	2018	No	
21	M	Whole body	Teeth 34, 48	^14^C, ^13^C	Water	2019	Yes	Yes
22	(M)	Cranium	Teeth 13	^14^C, ^13^C	Soil	2019	No	
23	M	Whole body, skeletonized	Teeth 35, 48	^14^C, ^13^C	Soil	2004	No	
24	NA	Mandible	Teeth 47	^14^C, ^13^C	Water	2019	No	
25	NA	Cranium	Teeth 17, 26, calvarium	^14^C, ^13^C	Water	2019	No	Yes, but failed
26	NA	bones from at least ^3^ persons	Teeth 17, 25	^14^C, ^13^C	Soil	2019	No	
27	NA	Cranium	Teeth 26, 36	^14^C, ^13^C	Air	2020	No	
28	M	Several bones	Teeth 16, 18	^14^C, ^13^C, AAR	In concrete	2020	No	Yes
29	NA	bones from at least ^2^ persons	Teeth 17	^14^C, ^13^C	Soil, plastic bags	2021	No	
30	M	Whole body	Teeth 35	^14^C, ^13^C	Water	2020	No	
31	M	Whole body	Teeth 47	^14^C, ^13^C	Water	2021	Yes	Yes
32	M	Several bones	Teeth 27	^14^C, ^13^C	Soil	2021	No	
33	M	Whole body	Teeth 43	^14^C, ^13^C	Soil	2003	No	
34	NA	Cranium	Teeth 37	^14^C, ^13^C	Air, indoor	2021	No	
		***Teeth not available*** ^4^						
35	NA	Cranium	Calvarium	^14^C, ^13^C	Air	2014	No	
36	F	Cranium and femur	Calvarium	^14^C, ^13^C	Water	2014	No	Yes, but failed
37	F	Cranium	Calvarium	^14^C, ^13^C	Air	2015	No	Yes
38	F	Cranium	Calvarium	^14^C, ^13^C	Air	2016	No	Yes, but failed
39	NA	Several bones	Calvarium	^14^C, ^13^C	Water	2015	No	Yes, but failed
40	NA	Several bones	Thoracic vertebra	^14^C, ^13^C	Water	2017	No	
41	M	Cranium	Calvarium	^14^C, ^13^C	Water	2017	No	Yes, but failed
14	M	Several bones	Calvarium	^14^C, ^13^C	Water	2017	Yes	Yes
42	F	Whole body	Femur	^14^C, ^13^C	Soil	2018	No	
43	NA	Several bones	Sacrum	^14^C, ^13^C	Water	2019	No	
44	NA	Several bones	Calvarium	^14^C, ^13^C	Water	2019	No	
45	NA	Several bones	Femur	^14^C, ^13^C	Soil	2019	No	
46	F	Cranium	Calvarium	^14^C, ^13^C	Air	2019	No	
25	NA	Cranium	Calvarium	^14^C, ^13^C	Water	2019	No	Yes, but failed
47	NA	Several bones	Humerus	^14^C, ^13^C	Water	2020	No	Yes, but failed
48	NA	Tibia, part of	Tibia	^14^C, ^13^C	Soil	2020	No	
49	NA	Several bones	Femur	^14^C, ^13^C	Soil	2021	No	
33	M	Whole body	Calvarium	^14^C, ^13^C	Soil	2003	No	
50	NA	Femur	Femur	^14^C, ^13^C	Soil	2021	No	
51	NA	Humerus	Humerus	^14^C, ^13^C	Water	2021	No	
52	NA	Humerus	Humerus	^14^C, ^13^C	Water	2021	No	

^1^ Brackets–sex was identified with conventional morphological skeletal markers. ^2^ In some cases, the find might have included additional bones that we were not informed about. ^3^ AAR–aspartic acid racemization. ^4^ Cases 14, 25, 33 teeth were analyzed.

**Table 2:** AMS F^14^C results for the tooth crowns and roots analyzed, and the estimated year of birth of the individuals.

Case	Sex	Tissue	Tooth	True DOB	Tooth Formation Time (yrs)	F^14^C	Error (2*σ*)	δ^13^C (‰)	Estimated Tooth DOB	Estimated Person DOB *	True DOD	D/L Ratio	Estimated Age (yrs)	Estimated DOD	Origin
1	F	Crown	16	1989.2	1.5	1.096	0.006	−17.1	NA	NA	2011.9	NA	NA		Sweden
1	F	Crown	17	1989.2	5.1	1.096	0.004	−16.6	1999.5	1994.4	2011.9	NA	NA		Sweden
1	F	Crown	46	1989.2	3.0	1.134	0.004	−17.8	1992.8	1989.8	2011.9	NA	NA		Sweden
1	F	Root	16	1989.2	6.8	1.096	0.004	−17.7	1999.4	NA	2011.9	NA	NA		Sweden
1	F	Root	17	1989.2	11.0	1.075	0.004	−18.0	2003.4	1992.4	2011.9	NA	NA		Sweden
1	F	Root	46	1989.2	6.7	1.101	0.004	−17.3	1998.4	1991.7	2011.9	NA	NA		Sweden
2	M	Crown	15	NA	4.8	1.216	0.007	−12.9	1984.3	1979.5	NA	NA	NA	2014 ± 5	NA
2	M	Crown	27	NA	5.1	1.202	0.007	−12.0	1985.6	1980.5	NA	NA	NA	2014 ± 5	NA
2	M	Root	15	NA	11.7	1.133	0.007	−12.9	1993.0	1981.3	NA	0.1122	36	NA	NA
2	M	Root	27	NA	12.4	1.130	0.007	−13.7	1993.3	1980.9	NA	0.1184	36	NA	NA
3	M	Crown	34	NA	4.1	0.970	0.004	−15.3	Pre-bomb	Pre-bomb	Pre-bomb	NA	NA	NA	Sweden
3	M	Crown	35	NA	5.3	0.959	0.004	−15.3	Pre-bomb	Pre-bomb	Pre-bomb	NA	NA	NA	Sweden
3	M	Root	34	NA	10.6	0.969	0.004	NA	Pre-bomb	Pre-bomb	Pre-bomb	NA	NA	NA	Sweden
3	M	Root	35	NA	11.0	0.966	0.004	NA	Pre-bomb	Pre-bomb	Pre-bomb	NA	NA	NA	Sweden
4	M	Crown	35	1992.7	5.3	1.107	0.010	−13.3	1997.4	1992.1	2012.0	NA	NA	NA	Poland
4	M	Crown	43	1992.7	3.6	1.099	0.009	−8.7	1998.9	1995.3	2012.0	NA	NA	NA	Poland
4	M	Root	43	1992.7	9.8	1.079	0.009	−9.4	2003.1	1993.3	2012.0	NA	NA	NA	Poland
5	M	Crown	13	1972.0	3.3	1.346	0.018	−13.2	1977.1	1973.8	2009.4	NA	NA	NA	Poland
5	M	Crown	35	1972.0	5.3	1.336	0.017	−12.7	1977.6	1972.3	2009.4	NA	NA	NA	Poland
5	M	Crown	47	1972.0	6.2	1.146	0.015	−13.5	1978.2	1972	2009.4	NA	NA	NA	Poland
5	M	Root	13	1972.0	10.2	1.201	0.016	−12.9	1986.2	1976	2009.4	NA	NA	NA	Poland
5	M	Root	35	1972.0	11.3	1.167	0.015	−13.4	1989.2	1977.9	2009.4	NA	NA	NA	Poland
5	M	Root	47	1972.0	12.2	1.323	0.016	−11.6	1992.0	1979.8	2009.4	NA	NA	NA	Poland
6	F	Crown	23	1975.4	3.3	1.283	0.013	−11.2	1980.4	1977.1	2014.6	NA	NA	2014.7	Poland
6	F	Crown	34	1975.4	4.1	1.275	0.013	−11.8	1980.9	1976.8	2014.6	NA	NA	2014.7	Poland
6	F	Crown	42	1975.4	3.2	1.299	0.016	−12.9	1979.2	1976	2014.6	NA	NA	2014.7	Poland
6	F	Root	23	1975.4	9.0	1.171	0.011	−11.9	1988	1979	2014.6	NA	NA	2014.7	Poland
6	F	Root	34	1975.4	9.3	1.171	0.011	−12.0	1988.4	1979.1	2014.6	NA	NA	2014.7	Poland
6	F	Root	42	1975.4	6.5	1.185	0.015	−12.5	1987.2	1980.7	2014.6	NA	NA	2014.7	Poland
7	NA	Crown	47	NA	6.2	1.013	0.009	−13.9	1956.0	1949.8	NA	NA	NA	NA	NA
7	NA	Crown	46	NA	3.0	0.996	0.009	−13.3	Pre-bomb	Pre-bomb	NA	NA	NA	NA	NA
7	NA	Root	47	NA	12.0	1.016	0.009	−13.5	1956.2	1944.2	NA	NA	NA	NA	NA
7	NA	Root	46	NA	7.0	1.012	0.009	−14.9	1956.2	1949.2	NA	NA	NA	NA	NA
8	M	Crown	14	1967.7	3.6	1.450	0.012	−15.0	1972.9	1969.3	2015.2	NA	NA	NA	Poland
8	M	Crown	17	1967.7	5.1	1.440	0.014	−14.9	1973.4	1968.3	2015.2	NA	NA	NA	Poland
8	M	Root	14	1967.7	10.9	1.260	0.012	−15.7	1981.6	1970.7	2015.2	NA	NA	NA	Poland
8	M	Root	17	1967.7	12.4	1.230	0.012	−16.1	1983.3	1970.9	2015.2	NA	NA	NA	Poland
9	M	Crown	13	NA	3.3	1.042	0.005	−13.0	1956.8	1953.5	NA	NA	NA	NA	NA
9	M	Crown	17	NA	5.1	1.053	0.007	−15.3	1956.9	1951.8	NA	NA	NA	NA	NA
9	M	Root	13	NA	9.6	1.280	0.007	−15.3	1962.0	1952.4	NA	NA	NA	NA	NA
9	M	Root	17	NA	11.7	1.516	0.008	−15.5	1963.0	1951.3	NA	NA	NA	NA	NA
10	(F)	Crown	14	NA	3.6	0.936	0.005	−6.0	Pre-bomb	Pre-bomb	NA	NA	NA	NA	NA
10	(F)	Crown	21	NA	2.2	0.941	0.005	−6.1	Pre-bomb	Pre-bomb	NA	NA	NA	NA	NA
10	(F)	Root	14	NA	9.7	0.997	0.005	−5.2	Pre-bomb	Pre-bomb	NA	NA	NA	NA	NA
10	(F)	Root	21	NA	8.3	0.992	0.005	−6.1	Pre-bomb	Pre-bomb	NA	NA	NA	NA	NA
11	(F)	Crown	13	NA	3.3	0.9800	0.003	−24.2	Pre-bomb	Pre-bomb	NA	NA	NA	Pre-bomb	NA
11	(F)	Crown	15	NA	4.8	0.988	0.003	−13.6	Pre-bomb	Pre-bomb	NA	NA	NA	Pre-bomb	NA
11	(F)	Root	13	NA	9.0	0.979	0.003	−12.9	Pre-bomb	Pre-bomb	NA	NA	NA	Pre-bomb	NA
11	(F)	Root	15	NA	10.7	0.985	0.003	−10.5	Pre-bomb	Pre-bomb	NA	NA	NA	Pre-bomb	NA
12	Na	Crown	34	NA	4.1	0.985	0.004	−13.2	Pre-bomb	Pre-bomb	NA	NA	NA	NA	NA
12	Na	Crown	38	NA	11.0	0.988	0.004	−13.2	Pre-bomb	Pre-bomb	NA	NA	NA	NA	NA
12	Na	Root	34	NA	10.0	0.982	0.004	−12.4	Pre-bomb	Pre-bomb	NA	NA	NA	NA	NA
12	Na	Root	38	NA	17.0	0.995	0.004	−12.1	Pre-bomb	Pre-bomb	NA	NA	NA	NA	NA
13	Na	Crown	16	NA	1.5	0.972	0.006	−14.9	Pre-bomb	Pre-bomb	NA	NA	NA	NA	NA
13	Na	Crown	24	NA	3.6	0.981	0.006	−15.4	Pre-bomb	Pre-bomb	NA	NA	NA	NA	NA
13	Na	Root	16	NA	1.5	0.971	0.007	−14.4	Pre-bomb	Pre-bomb	NA	NA	NA	NA	NA
13	Na	Root	24	NA	3.6	0.983	0.003	−14.3	Pre-bomb	Pre-bomb	NA	NA	NA	NA	NA
14	M	Crown	33	1965.2	3.6	1.550	0.008	−14.1	1969.4	1965.8	2008.6	NA	NA	NA	Finland
14	M	Crown	18	1965.2	11.5	1.379	0.006	−13.8	1975.5	1964.0	2008.6	NA	NA	NA	Finland
14	M	Root	33	1965.2	9.8	1.358	0.008	−14.6	1972.8	1963.0	2008.6	NA	NA	NA	Finland
14	M	Root	18	1965.2	17.0	1.251	0.006	−15.1	1978.4	1961.4	2008.6	NA	NA	NA	Finland
15	(M)	Crown	21	NA	2.2	0.903	0.004	−14.2	Pre-bomb	Pre-bomb	NA	NA	NA	NA	NA
15	(M)	Crown	38	NA	11.0	0.926	0.004	−12.8	Pre-bomb	Pre-bomb	NA	NA	NA	NA	NA
15	(M)	Root	21	NA	7.4	0.910	0.004	−13.3	Pre-bomb	Pre-bomb	NA	NA	NA	NA	NA
15	(M)	Root	38	NA	17.0	0.942	0.004	−12.4	Pre-bomb	Pre-bomb	NA	NA	NA	NA	NA
16	F	Crown	25	NA	4.8	0.980	0.004	−14.3	Pre-bomb	1945.2	1970.9	NA	25.7		NA
16	F	Crown	33	NA	3.6	0.990	0.004	−14.8	Pre-bomb	1927.4	1970.9	NA	43.5		NA
16	F	Crown	48	NA	11.0	0.980	0.004	−13.7	Pre-bomb	1924.7	1970.9	NA	46.2		NA
17	F	Crown	31	NA	1.9	1.432	0.006	−15.1	1973.5	1971.6	1995.2	NA	NA	NA	NA
17	F	Crown	34	NA	4.1	1.394	0.005	−13.9	1974.8	1970.7	1995.2	NA	NA	NA	NA
17	F	Root	34	NA	9.3	1.275	0.005	−14.6	1980.6	1971.3	1995.2	NA	NA	NA	NA
18	M	Crown	33	1960.8	3.6	1.313	0.004	−15.1	1962.7	1959.1	2015.6	NA	NA	NA	Vietnam
18	M	Crown	34	1960.8	4.1	1.516	0.004	−13.9	1963.1	1959.0	2015.6	NA	NA	NA	Vietnam
18	M	Crown	17	1960.8	5.1	1.582	0.004	−13.5	1963.2	1958.1	2015.6	NA	NA	NA	Vietnam
18	M	Root	33	1960.8	9.8	1.399	0.004	−15.1	1974.6	1964.8	2015.6	NA	NA	NA	Vietnam
18	M	Root	34	1960.8	10.6	1.384	0.004	−14.6	1975.2	1964.6	2015.6	NA	NA	NA	Vietnam
18	M	Root	17	1960.8	12.4	1.256	0.004	−15.3	1981.7	1969.3	2015.6	NA	NA	NA	Vietnam
19	M	Crown	24	NA	3.6	0.950	0.003	−13.2	Pre-bomb	Pre-bomb	NA	NA	NA	NA	NA
19	M	Crown	27	NA	5.1	0.964	0.003	−12.8	Pre-bomb	Pre-bomb	NA	NA	NA	NA	NA
20	NA	Crown	38	NA	11.0	0.900	0.003	−20.2	Pre-bomb	Pre-bomb	NA	NA	NA	NA	NA
21	M	Crown	34	2002.4	4.1	1.102	0.004	−13.9	2003.4	1999.3	2018.6	NA	NA	NA	Libya
21	M	Crown	48	2002.4	11.0	1.082	0.003	−15.5	2014.8	2003.8	2018.6	NA	NA	NA	Libya
21	M	Root	34	2002.4	10.6	1.074	0.004	−15.2	2003.5	1992.8	2018.6	NA	NA	NA	Libya
21	M	Root	48	2002.4	17.0	1.025	0.004	−13.7	2015.0	1998.0	2018.6	NA	NA	NA	Libya
22	(M)	Crown	13	NA	3.3	0.909	0.003	−13.8	Pre-bomb	Pre-bomb	NA	NA	NA	NA	NA
22	(M)	Root	13	NA	10.2	0.946	0.003	−13.8	Pre-bomb	Pre-bomb	NA	NA	NA	NA	NA
23	M	Crown	35	NA	5.3	1.127	0.003	−12.7	1957.8	1952.5	NA	NA	NA	NA	NA
23	M	Crown	48	NA	11.0	1.702	0.005	−12.9	1963.4	1952.4	NA	NA	NA	NA	NA
23	M	Root	35	NA	11.3	1.386	0.003	−13.2	1962.6	1951.3	NA	NA	NA	NA	NA
23	M	Root	48	NA	17.0	1.446	0.005	−13.3	1962.9	1945.9	NA	NA	NA	NA	NA
24	M	Crown	47	NA	6.2	0.809	0.003	−14.3	Pre-bomb	Pre-bomb	NA	NA	NA	NA	NA
24	M	Root	47	NA	12.2	0.809	0.003	−14.2	Pre-bomb	Pre-bomb	NA	NA	NA	NA	NA
25	NA	Crown	17	NA	5.1	0.772	0.003	−12.3	Pre-bomb	Pre-bomb	NA	NA	NA	NA	NA
25	NA	Crown	26	NA	1.5	0.789	0.003	−13.8	Pre-bomb	Pre-bomb	NA	NA	NA	NA	NA
25	NA	Root	17	NA	11.7	0.769	0.003	−20.7	Pre-bomb	Pre-bomb	NA	NA	NA	NA	NA
25	NA	Root	26	NA	7.1	0.769	0.003	−20.0	Pre-bomb	Pre-bomb	NA	NA	NA	NA	NA
26	NA	Crown	17	NA	5.1	0.989	0.004	−14.3	Pre-bomb	Pre-bomb	NA	NA	NA	NA	NA
26	NA	Crown	25	NA	4.8	0.961	0.003	−13.9	Pre-bomb	Pre-bomb	NA	NA	NA	NA	NA
26	NA	Root	17	NA	7.1	0.986	0.004	−15.1	Pre-bomb	Pre-bomb	NA	NA	NA	NA	NA
27	NA	Crown	26	NA	1.5	0.900	0.004	−19.8	Pre-bomb	Pre-bomb	NA	NA	NA	NA	NA
27	NA	Crown	36	NA	3.0	0.930	0.004	−19.7	Pre-bomb	Pre-bomb	NA	NA	NA	NA	NA
28	M	Crown	16	NA	1.5	1.144	0.005	−13.3	1991.8	1990.3	NA	0.077	34.3	2016-now	NA
28	M	Crown	18	NA	11.5	1.108	0.004	−13.4	1997.3	1985.8	NA	0.067	36.9		NA
29	(M)	Crown	17	NA	5.1	0.987	0.003	−16.7	Pre-bomb	Pre-bomb	Pre-bomb	NA	NA	Pre-bomb	NA
30	M	Crown	35	NA	5.3	1.127	0.004	−15.7	1994.0	1988.7	NA	NA	NA	NA	NA
30	M	Root	35	NA	11.3	1.056	0.004	−15.4	2007.4	1996.1	NA	NA	NA	NA	NA
31	M	Crown	47	1987.8	6.2	1.123	0.004	−14.8	1994.6	1988.3	2020.0	NA	NA	NA	Germany
31	M	Root	47	1987.8	12.2	1.063	0.004	−16.4	2005.9	1993.9	2020.0	NA	NA	NA	Germany
32	M	Crown	27	NA	5.1	0.897	0.003	−14.3	Pre-bomb	Pre-bomb	Pre-bomb	NA	NA	NA	NA
32	M	Root	27	NA	11.7	0.937	0.003	−13.7	Pre-bomb	Pre-bomb	Pre-bomb	NA	NA	NA	NA
33	M	Crown	43	NA	3.6	0.992	0.004	−12.2	Pre-bomb	Pre-bomb	NA	NA	NA	NA	NA
33	M	Root	43	NA	9.8	1.176	0.004	−13.1	1958.7	1949.9	NA	NA	NA	NA	NA
34	NA	Crown	37	NA	6.2	0.906	0.003	−12.8	Pre-bomb	Pre-bomb	prebomb	NA	NA	NA	NA
34	NA	Root	37	NA	12.0	0.904	0.003	−19.8	Pre-bomb	Pre-bomb	prebomb	NA	NA	NA	NA

* Estimated dob person (yrs) = estimated dob tooth (yrs)−tooth formation time (yrs).

**Table 3:** AMS F^14^C results for the bone samples. Levels above 1.0 imply that the person was alive after 1955. All values below 1.0 indicate that the person either was not alive after 1955 or died soon after that. All values represent analysis of cortical.

Case	Sex	Tissue	True DOB	F^14^C	Error (2*σ*)	δ^13^C (‰)	C/N	Estimated Living Period
14	M	Calvarium	1965.2	1.247	0.006	−20.9	3.3	>1955
14	M	Calvarium (trabecular)	1965.2	1.210	0.006	−21.8	3.3	>1955
14	M	Humerus	1965.2	1.209	0.006	−21.1	3.3	>1955
25	NA	Calvarium	NA	0.770	0.003	−20.6	3.2	Before 1955
33	M	Calvarium	NA	1.140	0.004	−27.7	3.3	>1955
35	NA	Calvarium	NA	1.040	0.008	−24.2	3.3	>1955
36	F	Calvarium	NA	1.003	0.009	−15.7	3.2	Before 1955
37	F	Calvarium	NA	0.979	0.006	−20.5	NA	Before 1955
38	F	Calvarium	NA	1.189	0.004	−21.6	NA	Before 1963
39	NA	Humerus	NA	0.693	0.003	−18.1	3.3	Before 1955
40	NA	Thoracic vertebra	NA	0.941	0.003	−18.1	NA	Before 1955
41	M	Calvarium	NA	1.216	0.006	−21.0	3.4	>1955
41	M	Calvarium (trabecular)	NA	1.259	0.006	−20.8	3.3	>1955
42	F	Femur	2009.9	1.054	0.004	−20.8	3.2	>1955
43	NA	Sacrum	NA	0.976	0.003	−18.9	3.2	Before 1900
44	NA	Calvarium	NA	0.913	0.003	−18.7	3.2	Before 1900
45	NA	Femur	NA	0.973	0.003	−19.4	3.2	Before 1955
46	F	Calvarium	NA	0.984	0.003	−19.4	3.3	Before 1900
47	NA	Humerus	NA	1.432	0.005	−20.1	NA	Before 1974
48	NA	Tibia	NA	0.991	0.004	−19.2	3.2	Before 1900
49	NA	Femur	NA	0.918	0.003	−19.1	3.2	Before 1900
50	NA	Femur	NA	0.994	0.003	−20.4	3.3	Before 1900
51	NA	Humerus	NA	0.789	0.003	−20.1	3.2	Before 1900
52	NA	Humerus	NA	0.854	0.003	−19.3	3.2	Before 1900

**Table 4:** Results of AAR analysis of teeth in four of the cases.

Case Information							Radiocarbon Analyses						Aspartic Acid Analyses		
Case	Sex	Tissue	Tooth No.	True DOB	True DOD	Tooth Formation Time	F^14^C	Error (2*σ*)	δ ^13^C ‰	Estimated Tooth DOB	Estimated DOB	Average DOB	D/L Ratio	Estimated Age	Estimated Year of Death
2	M	Crown	15	NA	NA	6.6	1.216	0.007	−12.9	1984.3	1977.7	1978.4			
2	M	Crown	27	NA	NA	6.5	1.202	0.007	−12.0	1985.6	1979.1				
2	M	Dentin	15	NA	NA	11.7	1.133	0.007	−12.9	1993,0			0.1122	36.0	2014 ± 5
2	M	Dentin	27	NA	NA	12.4	1.130	0.007	−13.7	1993.3			0.1184	36.0	2014 ± 5
14	M	Crown	33	1965.2	2008.6	3.6	1.550	0.008	−14.1	1969.4	1965.8	1964.9			
14	M	Crown	18	1965.2	2008.6	11.5	1.379	0.006	−13.8	1975.5	1964.0				
14	M	Dentin	33	1965.2	2008.6	3.6	NA	NA	NA	NA	NA		0.0433	36.8	2002.0
14	M	Dentin	43	1965.2	2008.6	3.6	NA	NA	NA	NA	NA		0.0428	36.6	
16	F	Crown	25	NA	1970.9	4.8	0.980	0.004	−14.3	Pre-bomb			NA		
16	F	Crown	33	NA	1970.9	3.6	0.990	0.004	−14.8	Pre-bomb			NA		
16	F	Crown	48	NA	1970.9	11.0	0.980	0.004	−13.7	Pre-bomb			NA		
16	F	Dentin	22	NA	1970.9	3.8	NA	NA	NA	NA	1945.2		0.0557	25.7	
16	F	Dentin	24	NA	1970.9	3.6	NA	NA	NA	NA	1924.7		0.0660	46.2	
16	F	Dentin	25	NA	1970.9	4.8	NA	NA	NA	NA	1927.4		0.0647	43.5	
28	M	Crown	16	NA	NA	1.5	1.144	0.005	−13.3	1991.8	1990.3	1988.0			
28	M	Crown	18	NA	NA	11.5	1.108	0.004	−13.4	1997.3	1985.8				
28	M	Dentin	16	NA	NA	1.5	NA	NA	NA	NA	NA		0.077	34.3	2016-today
28	M	Dentin	18	NA	NA	11.5	NA	NA	NA	NA	NA		0.067	36.9	

## Data Availability

All relevant data are provided in the article. More exact date of birth for subjects that were identified represent confidential information which is archived at the Swedish National Board of Forensic Medicine.
